# Cutoffs and cardiovascular risk factors associated with neck circumference among community-dwelling elderly adults: a cross-sectional study: Pontos de corte e fatores de risco cardiovascular associados com a circunferência do pescoço em idosos da comunidade: um estudo transversal

**DOI:** 10.1590/1516-3180.2016.0160110906

**Published:** 2016-06-03

**Authors:** Hélio José Coelho, Ricardo Aurélio Carvalho Sampaio, Ivan de Oliveira Gonçalvez, Samuel da Silva Aguiar, Rafael Palmeira, José Fernando de Oliveira, Ricardo Yukio Asano, Priscila Yukari Sewo Sampaio, Marco Carlos Uchida

**Affiliations:** I MSc. Physical Educator and Doctoral Student, Applied Kinesiology Laboratory, School of Physical Education, Universidade de Campinas (UNICAMP), Campinas (SP), Brazil.; II MSc. Physical Educator and Doctoral Stu­dent, Health Sciences Center, Universidade de Mogi das Cruzes (UMC), Mogi das Cruzes (SP), Brazil.; III Physical Educator and Master's degree Student, School of Physical Education, Universidade Católica de Brasília (UCB), Brasília (DF), Brazil.; IV PhD. Physical Educator and Adjunct Professor, School of Physical Education, Universidade Católica de Brasília (UCB), Brasília (DF), Brazil.; V PhD. Physical Educator and Postdoctoral Student, School of Arts, Sciences and Humanities, Universidade de São Paulo (USP), São Paulo (SP), Brazil.; VI PhD. Occupational Therapist and Researcher, Applied Kinesiology Laboratory, School of Physical Education, Universidade de Campinas (UNICAMP), Campinas (SP), Brazil.; VII PhD. Physical Educator and Head, Applied Kinesiology Laboratory, School of Physical Education, Universidade de Campinas (UNICAMP), Campinas (SP), Brazil.

**Keywords:** Anthropometry, Blood pressure, Obesity, Aged, Primary health care, Antropometria, Pressão sanguínea, Obesidade, Idoso, Atenção primária à saúde

## Abstract

**CONTEXT AND OBJECTIVE:**

In elderly people, measurement of several anthropometric parameters may present complications. Although neck circumference measurements seem to avoid these issues, the cutoffs and cardiovascular risk factors associated with this parameter among elderly people remain unknown. This study was developed to identify the cutoff values and cardiovascular risk factors associated with neck circumference measurements among elderly people.

**DESIGN AND SETTING:**

Cross-sectional study conducted in two community centers for elderly people.

**METHODS:**

435 elderly adults (371 women and 64 men) were recruited. These volunteers underwent morphological evaluations (body mass index and waist, hip, and neck circumferences) and hemodynamic evaluations (blood pressure values and heart rate). Receiver operating characteristic curve analyses were used to determine the predictive validity of cutoff values for neck circumference, for identifying overweight/obesity. Multivariate analysis was used to identify cardiovascular risk factors associated with large neck circumference.

**RESULTS:**

Cutoff values for neck circumference (men = 40.5 cm and women = 35.7 cm), for detection of obese older adults according to body mass index, were identified. After a second analysis, large neck circumference was shown to be associated with elevated body mass index in men; and elevated body mass index, blood pressure values, prevalence of type 2 diabetes and hypertension in women.

**CONCLUSION:**

The data indicate that neck circumference can be used as a screening tool to identify overweight/obesity in older people. Moreover, large neck circumference values may be associated with cardiovascular risk factors.

## RESUMO

**CONTEXTO E OBJETIVO:**

Em idosos, diversas ferramentas antropométricas podem apresentar complicações durante a mensuração. Embora a circunferência do pescoço pareça evitar tais problemas, os pontos de corte e fatores de risco cardiovascular associados a essa ferramenta em idosos permanecem desconhecidos. Este estudo foi desenvolvido para identificar os valores de ponto de corte e fatores de risco cardiovascular associados à circunferência do pescoço em idosos.

**DESENHO E LOCAL:**

Estudo transversal, realizado em dois centros comunitários para idosos.

**MÉTODOS:**

435 idosos (371 mulheres e 64 homens) foram recrutados. Os voluntários foram submetidos a avaliação morfológica (índice de massa corporal e cintura, quadril, e circunferência do pescoço) e hemodinâmica (valores da pressão arterial e frequência cardíaca). A análise pela curva *receiver operating characteristic* foi usada para determinar o valor preditivo dos valores de ponto de corte da circunferência do pescoço para identificação de sobrepeso/obesidade. Análise multivariada foi usada para identificar os fatores de risco cardiovascular associados com circunferência do pescoço larga.

**RESULTADOS:**

Os valores de corte para circunferência do pescoço (homens = 40,5 cm e mulheres = 35,7 cm) para detectar adultos idosos obesos, de acordo com o índice de massa corporal, foram identificados. Depois da segunda análise, circunferência do pescoço larga foi associada com elevado índice de massa corporal em homens e mulheres e elevados valores de pressão arterial, prevalência de diabetes *mellitus* tipo II e hipertensão em mulheres.

**CONCLUSÃO:**

Os dados indicam que a circunferência do pescoço pode ser utilizada como ferramenta de rastreio para identificar sobrepeso/obesidade em idosos. Ademais, altos valores de circunferência do pescoço podem estar associados com fatores de risco cardiovascular.

## INTRODUCTION 

Worldwide projections indicate that there will be an exponential increase in the geriatric population over the coming decades.^1^ Today, elderly people represent around 15% of the world population, but by 2050, this percentage is expected to double, to reach values near 30%.^1^ This phenomenon demands special attention, since aging is associated with increased cardiovascular risk factors (e.g. hypertension and type 2 diabetes), along with the risk of development of sarcopenia and frailty (e.g. decreased muscle power and strength).^2^^,^^3^^,^^4^^,^^5^

Epidemiological data from the World Health Organization (WHO) show that, worldwide, more than half a billion adults (i.e. > 18 years old) are overweight or obese.^6^ Among elderly people, obesity is one of the most prevalent morbidities, reaching approximately 40% of this population in some countries.^7^

Obesity is a multifactorial condition caused by social, economic, behavioral and biological factors. The prevalence rate of obesity has increased significantly over recent decades, both in developed and in developing countries, thus leading to pandemic status for this morbid condition.^8^^,^^9^ In Brazil, the prevalence of overweight among adults increased from 43% in 2006 to 52.5% in 2014. Among older adults, the prevalence of overweight and obesity is higher than among younger adults (18-34 years old).^10^

Obesity is associated with several forms of cancer (e.g. esophageal, colon and renal) and contributes towards development of many disorders: metabolic (e.g. glucose intolerance and hyperinsulinemia), hemodynamic (e.g. high blood pressure), endothelial (e.g. increased oxidative stress) and inflammatory.^8^^,^^9^ Moreover, obesity has been shown to correlate with physiological disorders (e.g. depression and anxiety), muscle weakness (i.e. sarcopenic obesity) and social and economic problems.^8^^,^^9^^,^^11^ Thus, obesity is a public health concern, and continuous monitoring and early detection can prevent the onset of its adverse outcomes.

Since the data and projections indicate that Brazilians are getting older and fatter, continuous monitoring is essential for avoiding these issues associated with obesity among older adults. Regarding the methods used to measure overweight/obesity, neck circumference (NC) has been suggested as a low cost, reliable, noninvasive, easy reproducible and efficient approach for identifying overweight and obese children, adolescents and adults, and cutoff values have been suggested for these populations.^12^^,^^13^^,^^14^^,^^15^^,^^16^^,^^17^^,^^18^^,^^19^^,^^20^^,^^21^Moreover, there is some evidence demonstrating that NC is associated with cardiovascular risk factors, such as high waist and hip circumferences, elevated blood pressure values and high total cholesterol and glycemia levels.^12^^,^^13^^,^^17^ However, even though optimal cutoff values for adults have been suggested and have been observed to be associated with cardiovascular risk factors (e.g. elevated blood pressure and insulin resistance),^20^^,^^21^ there are no data in the literature regarding specific cutoffs for the elderly population.

Therefore, the purpose of this study was to develop cutoff values for identifying overweight/obesity among older adults, and to indicate the cardiovascular risk factors associated with large NC values among women and men.

## OBJECTIVE

The aim of this study was to evaluate NC as a screening tool for detecting overweight and obesity in accordance with the body mass index (BMI) classification for the elderly population, as defined by chronology (≥ 60 years old); and secondarily to investigate the cardiovascular risk factors associated with large NC.

## METHODS

### Design

This study had a cross-sectional design and evaluated a convenience sample. Experiments were developed in the city of Poá, state of São Paulo, Brazil, in 2015.

## Subjects

The participants of the present study were recruited voluntarily from two specialized public community health centers for older adults in southeastern Brazil, between February and November 2015. In both centers, they undertook social activities that predominantly involved the physical and cognitive domains. The physical activities comprised a multimodal exercise program that stimulated several physical capabilities (muscle strength, muscle power and cardiorespiratory fitness) within the same exercise session. These sessions lasted approximately 40 minutes and involved low to moderate-intensity physical activities: water aerobics, folk dancing and yoga. Painting was the only activity that predominantly involved the cognitive domain. After registration, these older adults were automatically enrolled in all activities. However, they were not required to participate in or perform activities every day. It is important to mention that neither center was responsible for rehabilitation or for any kind of medical treatment, and that these elderly individuals were functionally and cognitively able to understand and perform all the activities. Moreover, for all participants with clinical diagnoses of diseases (e.g. hypertension or diabetes mellitus type II), their pathological condition was controlled and monitored by their private physician.

These volunteers were recruited according to convenience and were invited orally to participate in this research.

The inclusion criterion was that the subjects needed to be people aged 60 years or over who could perform the study procedures. The exclusion criteria were:

1. morbid obesity, according to the BMI classification (i.e. over 40 kg/m^2)^; and
2. presence of a disease or condition that could disturb the NC measurement (e.g. goiter).

All subjects provided informed consent before being enrolled in the study procedures. This study was approved by the Research Ethics Committee of the University of Mogi das Cruzes (Universidade de Mogi das Cruzes, UMC), and it was developed in accordance with the Declaration of Helsinki and with Resolution 196/96 of the Brazilian Health Council.

### Anthropometric measurements

### Weight, height and BMI

A weight scale with a Filizola (Brazil) stadiometer was used to measure body mass (kg) and height (cm). The BMI was determined as follows: body mass index = (kg)/(height [m])². A model proposed through the Health, Wellbeing and Aging (Saúde, Bem Estar e Envelhecimento, SABE) study in conjunction with the Pan-American Health Organization (PAHO) study was used to classify BMI.^22^ Values ≥ 28 kg/m² were considered to represent overweight/obesity for elderly adults.^22^

### Circumferences

**Figure 1:**
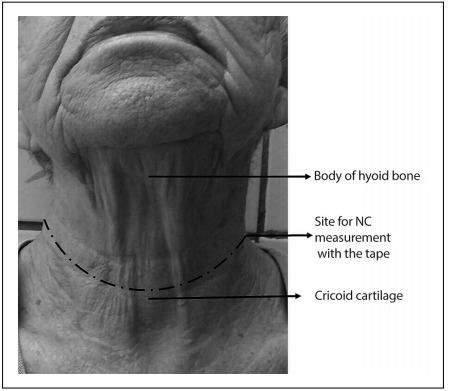
Neck circumference (NC) was measured just above the cricoid cartilage and perpendicular to the long axis of the neck. If necessary, to facilitate the measurement, the evaluator would possibly require the patient to look up. Starting from the small prominence of the thyroid cartilage, the evaluator applied light pressure using the forefinger and middle finger, moving downwards to find a small space and subsequently the cricoid cartilage in the region demonstrated in the photograph. The dashed line represents the point at which the NC should be measured. The cutoff values for NC evaluation were 35.7 cm for women and 40.5 com for men.

For all evaluations, i.e. waist circumference (WC), hip circumference (HC) and neck circumference (NC), a flexible and inextensible anthropometric tape (Sanny, Brazil) was used. The subjects remained in the standing position, with head held erect, eyes looking forward, arms relaxed at the side of the body and feet kept together, wearing light clothes. WC was assessed at the midpoint between the last floating rib and the highest point of the iliac crest.^23^ HC was evaluated at the highest point of the buttocks.^23^ NC was measured just above the cricoid cartilage and perpendicular to the long axis of the neck.^20^ If necessary, to facilitate the measurement, the evaluator would possibly require the patient to look up. Starting from the small prominence of the thyroid cartilage, the evaluator applied light pressure using the forefinger and middle finger, moving downwards to find a small space and subsequently the cricoid cartilage in the region demonstrated in Figure 1. All subjects were evaluated twice, and the larger measurement was used in the analyses.

### Prevalence of morbidities and use of medications

Information regarding prevalence of morbidities (pathological conditions) and use of medications was collected from the medical records of each subject by two researchers and the data were compared. Since both of the community health centers serve a large number of patients, and the medical team (i.e. nurse, physician and physical educator) is of limited size, the pathological conditions and medications used were simply recorded by the head physician and head nurse of each center. A specialist who was not affiliated to and was outside the center then made the diagnosis. In summary, before the participant began the activities in the center, a medical consultation was conducted and the diseases already diagnosed, along with the medications used, had to be informed by the physician. Moreover, over the course of the year, the participants in both centers underwent medical consultations and were asked about symptoms (e.g. pain, polydipsia or polyuria) that are associated with highly prevalent pathological conditions among elderly people (e.g. osteoarthritis or diabetes mellitus type II). Blood pressure and glycemia values were measured and recorded by the nurses on a personal card before all physical activities at any time during the day. It should be noted that the classification of cardiovascular disease (CVD) was based on previous clinical diagnoses of myocardial infarction, stroke (ischemic and hemorrhagic), heart failure (none of the subjects in the present study showed this), arrhythmia and heart valve problems. This personal card was presented by the patient to the physician on the day of the consultation, and, if necessary, the patient was referred to a specialist. If the diagnosis was confirmed, the patient needed to inform the nurse. Regarding the mean number of medications used by the patients (MNM), only the drugs associated with the disease(s) that had been clinically diagnosed were quantified. Therefore, over-the-counter medications were not included. Since the medical records were reviewed and updated every six months, the MNM related to the last six months.

### Cardiovascular parameters

All cardiovascular parameters were verified with the subjects at rest. The procedures for measuring blood pressure were adapted from the Seventh Joint National Committee for High Blood Pressure (JNC7).^24^ In summary, the elderly individual was placed in a sitting position on a comfortable chair for 15 minutes in a dark and quiet room. After this period, a cuff of the correct size was placed approximately at the midpoint of the upper left arm (heart level). An automatic, noninvasive and validated^25^ arterial blood pressure monitor (Microlife-BP 3BT0A, Microlife, Widnau, Switzerland) was used to measure systolic blood pressure (SBP), diastolic blood pressure (DBP) and heart rate (HR). The mean arterial pressure (MAP), double product (DP) and pulse pressure (PP) were evaluated according to the following equations:

MAP = [SBP + (2 * DBP)]/3; DP = SBP * HR; and PP = SBP − DBP.

The size of the arm cuff was selected after measuring the arm circumference (Sanny, São Paulo, Brazil).

### Statistical analyses

Phase one of the statistical analysis was performed to evaluated whether NC could be used as a screening tool for detecting conditions of overweight and obesity in accordance with the BMI classification among elderly women and men. Continuous data (e.g. age) and categorical data (e.g. prevalence) on the characteristics of these elderly adults according to BMI and gender were compared by means of the independent t test and chi-square test (χ²). Pearson's correlation was performed to analyze the association of NC with age and anthropometric measurements (i.e. BMI, WC, HC and WC/HC). Receiver operating characteristic (ROC) curve analyses were used to determine the predictive validity of NC, and to evaluate optimal cutoff values for identifying overweight/obese older adults in accordance with the BMI classification. Sensitivity and specificity were used to find the optimal cutoff values.

Phase two consisted of investigation of the cardiovascular risk factors associated with higher NC. After the subjects had been categorized dichotomously according to their NC values from phase one, continuous data were compared by means of the independent t-test. The χ² test was performed to investigated the association between the dependent categorical variable (i.e. NC) and independent categorical variables (age, BMI, SBP, DBP, MAP, HR, DP, PP, MNM, hypertension [HTN], diabetes mellitus type II [DMTII], arthritis and cardiovascular disease [CVD]). For the categorical determinations, cutoffs for the diagnosis of hypertension (in accordance with JNC7) were used for SBP and DBP, while medians were used for age, MAP, HR, DP, PP and MNM. Independent variables that showed P-values ≤ 0.20 were added sequentially one by one, in increasing P-value order, in the univariate logistic binary analysis. Similarly, variables that showed P-values ≤ 0.05 were added sequentially, one by one, in increasing order of the value of P in the multivariate logistic binary analysis. The fit of the multiple regression model was evaluated by means of the Hosmer-Lemeshow^26^ test. All analyses were conducted using the Statistical Package for the Social Sciences software (SPSS; IBM, Chicago, IL, USA), version 20.0.

## RESULTS

Firstly, 758 subjects from two specialized public community health centers were invited to participated in the present study. Two hundred and thirty-four of the invited subjects were middle-aged and unable to commit to the present study. Among the remaining 524 older adults, 76 refused to participate, six did not sign the informed consent statement and seven were excluded due to morbid obesity (n = 5) or goiter (n = 2). Therefore, 435 volunteers (371 women [85.2%] and 64 men [14.8%]), of whom 396 were from the first center (Cantinho de Convivência do Idoso da Cidade de Poá) and 39 were from the second center (Instituto Renascer), formed the sample of the present study.

The prevalences of overweight and obesity (after exclusion of the morbidly obese individuals) were 44.8% (women) and 46.6% (men), according to the BMI classification. Table 1 shows the characteristics of the sample according to BMI and gender.

**Table 1:**
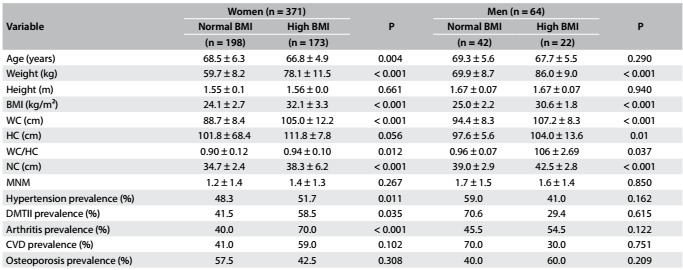
Characteristics of the older adults according to BMI and gender

BMI = body mass index; WC = waist circumference; HC = hip circumference; WC/HC = waist to hip ratio; NC = neck circumference; DMTII = diabetes mellitus type II; CVD = cardiovascular disease; MNM = mean number of medications used by the patients.

The anthropometric measurements (i.e. WC, HC, WC/HC and NC) were higher in high-BMI subgroups than in low-BMI subgroups, in all groups (P = 0.000). The prevalences of HTN, DMTII and arthritis were higher in high-BMI subgroups than in low-BMI subgroups among the elderly women, but not among the elderly men. The prevalences of MNM, CVD and osteoporosis did not differ between the subgroups.

Pearson's correlation was performed between NC (dependent variable) and age, weight and anthropometric measurements (e.g. WC, HC and WC/HC) (independent variables). NC was shown to be significantly correlated with age, weight, BMI, WC and WC/HC, in both groups (men and women). However, NC did not show any significant correlation with HC in either group (men: r *=* 0.20; P = 0.11; women: r *=* 0.02; P = 0.73). Moreover, Pearson's classification was similar in both groups. Age and WC/HC showed weak correlation coefficients (Age: men: r *=* -0.15; women: r *=* -0.12; and WC/HC: men: r *=* 0.11*;* women: r *=* 0.02); while weight (men: r *=* 0.67*;* women: r *=* 0.56), BMI (men: r *=* 0.55; women: r *=* 0.47) and WC (men: r *=* 0.60; women: r *=* 0.45) showed moderate correlation coefficients.

The receiver operating characteristic (ROC) curve, optimal cutoff and negative and positive likelihood ratio (LR) results are described below. The area under the curve (AUC) showed values higher than 0.7 in both groups (men = 0.817 and women = 0.819), thus indicating clinical significance. Optimal cutoffs were found both for men (40.5 cm; CI: 0.704-0.929; P < 0.001; sensitivity: 0.864; and specificity: 0.317) and for women (35.7 cm; CI: 0.774-0.863; P < 0.001; sensitivity: 0.821; and specificity: 0.311). The results demonstrated that elderly men (LR+: 2.724; LR-: 0.367) and women (LR+: 2.637; LR-: 0.379) who presented values higher than the optimal cutoff for NC were approximately twice as likely to be overweight and obese according to the BMI classification as were older adults with small NC values.

Because of the results from the first phase, which demonstrated the capacity of NC to screen for overweight and obesity among elderly people of both genders in accordance with their BMI, a second analysis was performed to ascertain the factors associated with large NC. After excluding one volunteer (due to missing data), the sample (n = 434) was allocated to four groups according to NC (i.e. large or small) and gender (i.e. men or women). Weight and BMI were higher in the large-NC subgroup than in the normal-NC subgroup, regardless of gender. Blood pressure measurements at rest did not show similar behavior between the groups. The large-NC subgroup of elderly women showed higher DBP and MAP than did the normal-NC subgroup. Meanwhile, the large-NC subgroup of elderly men showed higher PP than did the normal-NC subgroup. These data are shown in Table 2.

**Table 2:**
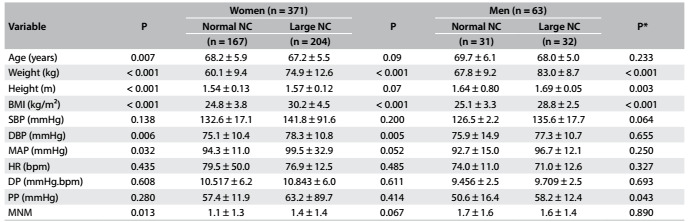
Characteristics of the older adults according to NC and gender

NC = neck circumference; BMI = body mass index; SBP = systolic blood pressure; DBP = diastolic blood pressure; MAP = mean arterial pressure; HR = heart rate; DP = double product; PP = pulse pressure; MNM = mean number of medications used by the patients. *P-value refers to the comparison among the Large NC (women and men) and Normal NC (women and men).

A χ² test was performed to analyze the association between categorical variables. Among the elderly women, age (0.028), BMI (< 0.001), MAP (0.005), HTN (0.001), DMTII (< 0.001) and arthritis (0.041) showed significant P-values to be added to the logistic binary analyses. In turn, the elderly men presented a lower number of associated variables than the elderly older women: BMI (< 0.001), SBP (0.056), MAP (0.055), HTN (0.030) and arthritis (0.023)

Tables 3 and ^4^ show the results regarding the unadjusted odds ratio (OR), adjusted OR and 95% CI for NC in both groups (women and men, respectively).

**Table 3:**
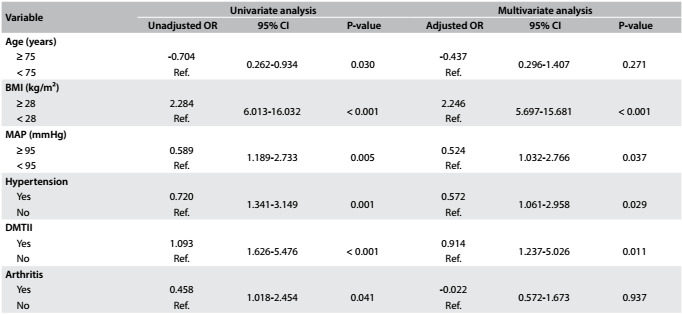
Unadjusted odds ratio (OR), adjusted OR and 95% confidence intervals (CI) for neck circumference (NC) among older women

BMI = body mass index; MAP = mean arterial pressure; DMTII = diabetes mellitus type II; CVD = cardiovascular disease; Ref = reference.

**Table 4:**
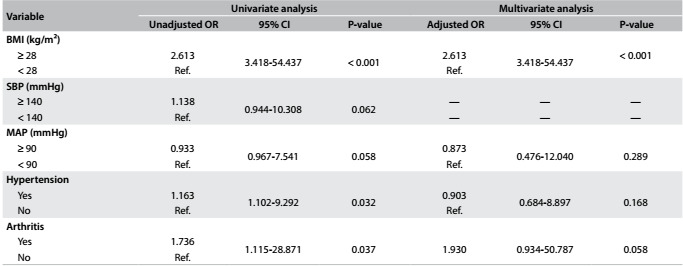
Unadjusted odds ratio (OR), adjusted OR and 95% confidence intervals (CI) for neck circumference (NC) in older men

BMI = body mass index; SBP = systolic blood pressure; MAP = mean arterial pressure; Ref = reference.

After the unadjusted OR analyses, age (P = 0.03), BMI (P < 0.001), MAP (0.005), hypertension (P = 0.001), DMTII (P < 0.001) and arthritis (P = 0.04) were added sequentially, one by one, in increasing P-value order, in the multivariate analysis on the group of elderly women. Likewise, BMI (P < 0.001), SBP (P = 0.06), MAP (P = 0.05), HTN (P = 0.03) and arthritis (P = 0.03) were added in the analysis on the group of elderly men. After the adjusted OR analyses, BMI was still found to be significantly associated with large NC among the elderly women (adjusted OR: 2.246 [5.697-15.681]) and elderly men (adjusted OR: 2.613 [3.418-54.437]). However, DMTII (adjusted OR: 0.914 [1.237-5.026]), high MAP (adjusted OR: 0.524 [1.032-2.766]) and HTN (adjusted OR: 0.572 [1.061-2.958]) also showed significant results in the group of elderly women.

## DISCUSSION

In this study, NC was shown to be a useful screening tool for verifying conditions of overweight/obesity through the BMI among elderly women and men, since AUC showed values higher than 0.7 in both groups. Furthermore, these AUC values were higher in relation to NC than in relation to other parameters commonly used to assess body composition, such as WC/HC (AUC: men = 0.68; and women = 0.62). After identifying the cutoff values for NC (men = 40.5 cm; and women = 35.7 cm) (Figure 1), a second analysis was performed to ascertain the cardiovascular risk factors associated with large NC. The data showed that large NC was associated with high BMI in both groups. Moreover, large NC among elderly women was also associated with high MAP and high prevalences of HTN and DMTII.

These data seem to be important, given that some authors have suggested that NC is a low-cost, reliable, noninvasive and easy reproducible tool.^12^^,^^13^^,^^17^ Furthermore, some parameters that are usually used to evaluate obesity (e.g. waist circumference) can be burdensome to use because of the necessity for extensive training of the evaluators and because of measurement site differences, clothing restrictions, religious and social factors, time taken and weather conditions.^14^^,^^18^^,^^19^^,^^27^ These factors may preclude or at least decrease the feasibility of using other parameters (WC and HC).^14^^,^^18^^,^^19^^,^^27^ On the other hand, these issues seem to be avoided during NC measurement.^14^^,^^18^^,^^19^^,^^27^

Moreover, it is possible to suggest that NC may be a more suitable means for anthropometric measurements among elderly people than other tools, since its evaluation can be performed with the subject in a seated position, with no need to use light clothing (thereby avoiding awkward moments). In addition, the breathing phase does not change the results. Indeed, older adults may present conditions that impair their capacity to remain in an upright standing position, such as frailty, sarcopenia, osteopenia, osteoporosis, arthritis or different kinds of pain or skeletal muscle weakness. Elderly people may have impaired ability to maintain breathing control (e.g. in cases of chronic obstructive pulmonary disease). They may have religious and/or other beliefs regarding clothing, such as the necessity to wear a dress or skirt during measurements, especially among older women.

Studies have been conducted to describe NC cutoff values for factors associated with metabolic syndrome among Brazilian adults.^20^^,^^21^ However, to our knowledge, our study was the first to ascertain cutoff values for NC in relation to obesity among elderly Brazilians. Thus, because of the lack of studies that aimed to record the association between NC and cardiovascular risk factors among elderly Brazilians, the present discussion is based in both of these studies, which studied samples with a wide age range (i.e. approximately 18-74 years of age).^20^^,^^21^ Interestingly, both of these studies show cutoff values similar to those of our study.^20^^,^^21^ Data from Stabe et al. showed that men and women with NC values larger than 40 cm and 36.1 cm, respectively, had higher odds of presenting show insulin resistance and metabolic syndrome than did adults with small NC.^21^ Similarly, Baena et al., who studied a sample of 8726 Brazilian adults, showed that men and women with large NC (> 40 cm and > 34.1 cm, respectively) had higher odds of presenting insulin resistance, low HDL, elevated blood pressure and high triglycerides than did the small-NC group.^20^

Regarding the results from correlating NC with cardiovascular risk factors, other studies conducted using Brazilian samples^20^^,^^21^^,^^28^^,^^29^ and non-Brazilian samples^12^^,^^13^^,^^17^ corroborate our data. These studies showed that NC is positively associated with fasting triglyceride, glucose, insulin, adiponectin, glycated hemoglobin and blood pressure values, carotid intima-media thickness, uric acid levels and plasminogen activator inhibitor levels (PAI-1), as well as negatively with HDL-cholesterol values.^12^^,^^13^^,^^17^^,^^20^^,^^21^^,^^28^^,^^29^

Interestingly, even though the HTN condition was associated with large NC among elderly women, the hemodynamic measurements made at rest in the present study, except for MAP among elderly women, did not show any association with NC. In both community centers, the elderly individuals underwent constant medical monitoring, since blood pressure was measured every day and abnormalities in these measurements were reported immediately to the head nurse. If these measurements were recurrent, the patients would if necessary be referred to a specialist (i.e. a cardiologist). Taken together with data on the MNM (approximately 1.5), this may indicate that the absence of any association between the factors (hemodynamic measurements at rest and NC) was due to control over the clinical parameters (i.e. blood pressure and heart rate) that are associated with manifestations of the pathological condition (i.e. HTN). However, despite the use of automatic, noninvasive and validated arterial blood pressure monitoring,^25^ blood pressure is not a sensitive hemodynamic measurement and better tools, such as ambulatory blood pressure monitoring (ABPM) need to be used to make comparisons with our data and provide better understanding about this association.

Even though the present study was not able to elucidate the mechanisms that can explain the association of NC with diabetes and HTN, evidence in the literature allows inferences to be made. As mentioned earlier, cross-sectional studies have been showing that NC is associated with factors present in the genesis of DMTII, such as high fasting glucose, insulin and adiponectin, and in the progression of DMTII (i.e. glycated hemoglobin).^12^^,^^13^^,^^17^^,^^28^^,^^29^ Similarly, increased carotid intima-media thickness, which is a marker of atherosclerosis and, consequently, associated with hypertension, has been shown to have an association with large NC.^29^

Moreover, it has been suggested that NC is a surrogate marker for upper-body subcutaneous fat,^20^^,^^21^^,^^28^^,^^29^ which is more lipolytically active than lower body fat,^30^^,^^31^ due to its association with insulin resistance, impaired glucose uptake, atherosclerosis and endothelial dysfunction. Therefore, higher bioavailability of free fatty acids can be suggested as a common pathway in the relationship between NC and DMTII/HTN.

Some of the limitations of this study include

- the lack of biochemical measurements, which would have had the capacity to lead to better understanding about the association of NC with cardiovascular risk factors among elderly people;
- the limited number of men participating in the research protocol, which may represent bias and limitation on the inferences possible;
- use of data in the medical files to quantify the prevalence of morbidities and drug consumption; and
- lack of cutoffs for abnormal NC.

Thus, a study design comprising large numbers of non-selected samples and follow-up in order to identify individuals who develop major clinical conditions is necessary. Therefore, further studies evaluating larger samples and including biochemical measurements are recommended in order to confirm our findings.

## CONCLUSION

Neck circumference is a useful screening tool for detecting overweight/obesity among community-dwelling older adults. Furthermore, it was verified that large NC is associated with increased cardiovascular risk factors.
